# Vocabulary Learning in Chinese as a Second Language: Exploring the Role of Self-Regulation in Facilitating Vocabulary Knowledge of Second Language Learners

**DOI:** 10.3389/fpsyg.2022.893900

**Published:** 2022-07-15

**Authors:** Sida Zhu, Cong Wang

**Affiliations:** Department of Chinese Language Studies, The Education University of Hong Kong, Tai Po, Hong Kong SAR, China

**Keywords:** vocabulary knowledge, breadth and depth, self-regulation, Vietnamese university students, Chinese vocabulary

## Abstract

Vocabulary knowledge comprises depth and breadth, which are regarded as important indicators of second language (L2) learning capability. Self-regulation is a key factor in promoting vocabulary knowledge. However, the role and contribution of depth and breadth in and to L2 learning, as well as the predictive role of different factors of self-regulation in depth and breadth, remain unclear. Therefore, this study aims to identify the relationship between vocabulary knowledge and self-regulation by establishing a structural equation model based on exploratory factor analysis (EFA) and confirmatory factor analysis (CFA) using self-regulation and vocabulary knowledge (depth and breadth) questionnaires. A total of 215 Vietnamese university students participated in the research. The results show that Vietnamese university students generally obtain high scores in breadth, but their scores vary in depth, which indicates although most of them can accurately understand some aspects of Chinese word meaning, they are not able to command the form and usage of words. In addition, there is a negative correlation between self-regulation and vocabulary breadth, which demonstrates that high self-regulation, especially emotional control, can affect Vietnamese university students’ vocabulary learning. This study also proposes some suggestions for Chinese vocabulary teaching.

## Introduction

Vocabulary knowledge plays a significant role in learning Chinese as a second language (CSL) and is considered an essential indicator of second language (L2) learning capability (Nation, 2011; Webb and Sasao, 2013; Matthews, 2018; Zhou, 2021). Self-regulation is a dynamic concept that represents individuals’ involvement in learning, including cognitive, affective, behavioral, and motivational aspects that encourage learners to actively modify their personal learning goals in response to external changes to achieve desirable outcomes (Zimmerman and Risemberg, 1997; Dörnyei, 2005). As an essential factor, self-regulation impacts the overall improvement of L2 learning (Oxford, 2011; Gunning and Oxford, 2014; Ma and Oxford, 2014). A previous study found a correlation between self-regulation and L2 development, especially in the development of learners’ vocabulary; to be precise, L2 learners’ vocabulary knowledge was found to predict improvement in self-regulation (Rasekh and Ranjbary, 2003; McClelland et al., 2007; Vallotton and Ayoub, 2011; Seker, 2015). Researchers have also suggested that there is possible bidirectionality between the two factors (Fischer and Bidell, 2006). For example, research on Vietnamese university students’ vocabulary levels and self-regulation showed that self-regulation can improve students’ vocabulary levels and that the improvement of vocabulary levels can further improve students’ self-regulation levels (Truong and Wang, 2019; Kim et al., 2021). However, other researchers have suggested that excessive self-regulation may negatively affect Vietnamese university students’ vocabulary knowledge (Phuong, 2017; Vu and Peters, 2020).

The variances in the relationship between self-regulation and language capability in these studies may be affected by learners’ language levels, including vocabulary levels (August et al., 2009; Bohlmann et al., 2015). After comparing learners with different levels of language capability, researchers found that middle- and low-level L2 learners use self-regulation more frequently and that self-regulation is an essential indicator in predicting their L2 learning performance (Seker, 2015). Previous studies have mostly focused on the classification of vocabulary knowledge, such as depth and breadth, and measuring L2 learners’ performance in vocabulary knowledge (Nation, 1983, 2020; Nation and Beglar, 2007; Schmitt, 2010; Webb and Nation, 2017; Fan, 2020). They have not investigated whether there is dissimilarity at different levels of learners’ vocabulary depth and breadth, the possible causes of these variances, or the correlation between these variances and vocabulary learning capability. In addition, previous studies have only shown a correlation between self-regulation and vocabulary knowledge (Bodrova and Leong, 2007; Raver et al., 2011; Naderifar, 2018). However, there is no in-depth explanation of this correlation, i.e., whether the correlation is bidirectional, single, or overlapping. Furthermore, if self-regulation predicts vocabulary depth and breadth, the ways in which different self-regulation factors are associated with depth and breadth have not been explored. To clarify the confusion, this study adopted a quantitative research method, with the use of questionnaires involving self-regulation and the depth and breadth of vocabulary knowledge, to explore L2 learners’ performance on the depth and breadth of vocabulary knowledge by investigating 215 Vietnamese university students. The study also analyzed the relationship between different self-regulation factors and vocabulary knowledge (depth and breadth). This study aims to answer the following research questions:

1. How does vocabulary knowledge (depth and breadth) reflect the vocabulary learning of Vietnamese university students in CSL?
2. What are the relationships among different internal factors of self-regulation in vocabulary learning?
3. Which factor(s) of self-regulation predict(s) vocabulary knowledge (depth and breadth)?

## Literature Review

### The Impact of Self-Regulation on Second Language Learning

Self-regulation includes cognitive, metacognitive, and different dimensions of social behavior (Oxford, 2011; Zimmerman, 2011). It is an active, multidimensional process through which learners can set learning goals and actively motivate, regulate, and maintain their emotions and learning behaviors and eventually contribute to improving their learning performance (Dörnyei, 2005; Tseng et al., 2006). Self-regulation enhances language interaction among L2 learners and facilitates their adaptation to the classroom learning environment (Bohlmann and Downer, 2016; DiBenedetto, 2018; Yerdelen and Sungur, 2019). Learners with higher self-regulation capabilities (SRCs) are more focused on tasks and less distracted by external factors, such as early school experiences, classroom organization, and schooling effects. They can also correct deficits in the L2 learning process (Morrison et al., 2010; Cadima et al., 2019; Manchón, 2020). Furthermore, teachers may identify potential factors that influence L2 learning by determining their degree of self-regulation (Phan and Locke, 2015).

Self-regulation improving L2 learning has been supported by various studies (Oxford, 2011; Gunning and Oxford, 2014; Ma and Oxford, 2014; Hromalik and Koszalka, 2018; Abdolrezapour and Ghanbari, 2021). For example, L2 learners actively participate in activities through the appropriate use of self-regulation in classroom learning, and they also adequately practice what they have acquired in daily life to improve their speaking, writing, and reading outside of the classroom (Andrade and Evans, 2012; Ma and Oxford, 2014; Yabukoshi, 2021; Tian et al., 2022). Different models have been constructed to discuss the interactions between self-regulation and variables such as motivation, learning effectiveness, and time in previous research (Stoeger et al., 2014; Vanslambrouck et al., 2019), and these interactions are affected by personal effort and vary irregularly (Nietfeld et al., 2014; Vanslambrouck et al., 2019). Taking Vietnamese university students as an example, when facing difficulties in L2 learning, learners with higher language capability perform significantly better concerning self-regulation than other students (Kim et al., 2021). More importantly, Vietnamese university students with lower L2 capability have more initiative and reduce their negative emotions in L2 learning (Truong and Wang, 2019; Kim et al., 2021).

### Vocabulary Knowledge (Depth and Breadth) and Its Contribution to Second Language Capability

Vocabulary knowledge is a complex structure involving multiple components (González-Fernández, 2022). Earlier research focused more on the form and meaning of vocabulary knowledge and discussed the connections between them (Melka, 1997). However, these studies did not concern the multidimensional nature of vocabulary. They also did not explore the roles of different components in vocabulary knowledge or the contribution of each component to vocabulary knowledge. The complex structure makes it challenging to measure vocabulary knowledge (González-fernández and Schmitt, 2020). The Vocabulary Size Test and the Word Associates Test have been proposed to address the different dimensions of vocabulary knowledge (Nation, 1986, 2020; Nation and Beglar, 2007; Schmitt, 2010; Webb and Nation, 2017; Fan, 2020). Previous research has proposed that vocabulary knowledge should include depth and breadth to conceptualize the different dimensions of vocabulary knowledge. Depth represents the extent to which learners are aware of the form, meaning, and usage aspects of L2 vocabulary, while breadth relates to word forms and primary meanings, which are also known as form-meaning associations (Stæhr, 2009; Webb, 2013; Zhang and Lu, 2015; Luo et al., 2021; Teng, 2021). However, these two aspects are inseparable. Breadth indicates L2 learners’ familiarity with the number of target words and is recognized as the main component of vocabulary knowledge, while depth covers breadth to a certain extent; thus, one cannot be separated from the other (Qian and Lin, 2020; Yanagisawa and Webb, 2020).

In addition, numerous studies have found a high correlation between depth and breadth (Nation, 1983; Schmitt et al., 2001; Nation and Beglar, 2007; Zhang and Lu, 2015; Fan, 2020). For example, Thi and Nation (2011) found that the improvement in vocabulary breadth promoted depth growth among Vietnamese university students and that the correlation between this phenomenon and the L2 level of Vietnamese university students was positive. However, such a “positive correlation” was only found in some L2 vocabulary as the study progressed. For example, participation in additional listening and speaking vocabulary training improved Vietnamese university students’ L2 vocabulary learning and deepened their understanding of the form and meaning of words. Nevertheless, no significant correlation has been found between increased awareness and new vocabulary (Vu and Peters, 2020). It has been found that the strong relationship between vocabulary depth and breadth is influenced by L2 learners’ language capability, especially vocabulary level and that such variability expands in subsequent L2 learning (Webb, 2007; Chen and Truscott, 2010; Schmitt, 2014; Wang and Treffers-Daller, 2017; Puimège and Peters, 2019). Vocabulary knowledge can affect L2 learning, but the amount of contribution and the degree of influence of different dimensions of vocabulary knowledge, such as depth and breadth, on L2 learning remain to be further analyzed.

### The Role of Self-Regulation in Vocabulary Knowledge

Learners with better SRCs benefit more from L2 learning than others do (Bodrova and Leong, 2007; Raver et al., 2011; Naderifar, 2018). Among Vietnamese university students, there is a high correlation between their self-regulation and their vocabulary knowledge; furthermore, vocabulary knowledge, and self-regulation can influence each other and contribute to the improvement of those students’ L2 vocabulary levels (Truong and Wang, 2019). However, it has been suggested that this increase exists only in the early and middle stages of learning a new language. Researchers have found no significant predictive effect between self-regulation at other stages and vocabulary knowledge; in contrast, L2 learners have shown negative emotions and reduced personal learning efficiency (Weiland et al., 2014; Connor et al., 2016; ten Braak et al., 2019). Compared to L2 learners from other countries, Vietnamese university students have been shown to perform much worse on self-regulation (Kim et al., 2021). The newly enrolled university students performed even worse regarding self-regulation than those with extensive learning experience (Wang and Bai, 2017). There is no significant correlation between self-regulation and vocabulary knowledge for some L2 learners due to external factors such as family income and parental influence (Fuhs and Day, 2011; von Suchodoletz et al., 2011; Mathis and Bierman, 2015; Fung et al., 2019; Ramsook et al., 2020). Other studies have also found that self-regulation is not influenced by other factors, while a significant direct effect has been found between L2 attitudes toward learning and vocabulary knowledge (Yeşilbursa and Bilican, 2013; Su et al., 2019; Lan et al., 2020). In addition to attitudes, factors such as the number of years of L2 learning, the duration of learning, and self-regulation among Vietnamese university students also have significant impact on vocabulary knowledge.

Other researchers have used the strategic self-regulation model to classify self-regulation into different dimensions. Data from questionnaires have thus been analyzed to examine L2 learners’ use of self-regulation (Oxford, 2011; Seker, 2015). Unfortunately, the structural relationship between the different factors has not been strictly differentiated, which has resulted in overlapping factors (Seker, 2015). Especially for Vietnamese university students with varying vocabulary levels, the gap in their performance on self-regulation depending on their language capability has been found to be large (Truong, 2017). Through longitudinal studies, researchers have found that some factors, such as learning time, affect the predictive effect between self-regulation and vocabulary knowledge (Fuhs et al., 2014; Weiland et al., 2014; Hromalik and Koszalka, 2018; Birgisdottir et al., 2020). Gender has also been considered a significant factor that accounts for the variance in the relationship between self-regulation and vocabulary knowledge in Vietnamese university students (Truong and Wang, 2019). However, the number of female students usually outweighs that of male students at university, which results in a great disparity in gender being present in previous studies (Truong, 2017; Truong and Wang, 2019). Furthermore, as the facilitative effect of self-regulation on L2 vocabulary is limited to the beginning of vocabulary learning, some self-regulation factors fail to predict learners’ language skills (Cadima et al., 2019). Some self-regulation factors, such as emotion regulation, are significantly correlated to Chinese vocabulary learning strategies and vocabulary knowledge among Vietnamese university students. However, other self-regulation factors are not significantly correlated (Wilang and Duy, 2021).

In general, vocabulary knowledge (depth and breadth) plays an essential role in Vietnamese university students’ Chinese language learning. However, previous research has not yet analyzed the relationship between vocabulary depth and breadth and Vietnamese university students’ Chinese language capability. Although some studies have indicated that self-regulation influences the process of Chinese vocabulary learning among Vietnamese university students, the predictive role of self-regulation factors in vocabulary knowledge (depth and breadth) has not been analyzed. Therefore, a deep dive into the relationship between different factors and vocabulary depth and breadth deserves attention from researchers.

## Materials and Methods

### Participants

A total of two hundred and fifteen L2 Chinese learners who were juniors and seniors majoring in the Chinese language from a Vietnamese university participated in this study. Vietnamese was the native language of all participants. Some eleven participants who did not complete the questionnaires on time were excluded. The final data included 204 students (females = 154, males = 50) between the ages of 18 and 24. The imbalance in the gender ratio of participants is mainly due to the large disparity between male and female students majoring in the Chinese language at the university. All participants were informed in advance that the study was anonymous and voluntary and that participation would not impact their academic performance. In addition, this study did not involve any personal privacy questions, and all data were kept strictly confidential and used for research purposes only.

The participants were selected according to the following two criteria. First, all participants needed to complete the *Chinese vocabulary course* before data collection for the study. The *Chinese vocabulary course*, which focuses on the pronunciation, form, meaning, and usage of Chinese vocabulary, is a compulsory course for students majoring in the Chinese language at the university. The whole course lasts for 30 h throughout the first semester of students’ junior year, with the aim of facilitating Vietnamese students to use Chinese vocabulary precisely in their communication. Second, the participants were required to be junior and senior university students rather than freshmen and sophomores because after taking the *Chinese vocabulary course*, students will have mastered an abundant amount of Chinese vocabulary. Such mastery enables them to distinguish Chinese synonyms and antonyms, as well as to form sentences using those synonyms and antonyms, which is exactly the important content this study focused on in the utilized depth and breadth questionnaire. Freshmen and sophomores who have not received systematic training in Chinese vocabulary and have not learnt Chinese for enough time were excluded from the study because the depth and breadth questionnaire might be too demanding for them.

### Instrument

Two questionnaires were used in this study. Questionnaire 1 (Q1), which was used to investigate the participants’ self-regulation, used a 5-point Likert scale consisting of “1” = “*never*,” “2” = “*rarely*,” “3” = “*sometimes*,” “4” = “*often*,” and “5” = “*always.*” Questionnaire 2 (Q2) was adopted to examine the depth and breadth of the participants’ Chinese vocabulary knowledge; this questionnaire was multiple choice and required the participants to choose one correct answer from four given options. All items in Q1 and the items in Q2 concerning depth were written in both Chinese and Vietnamese. However, the items involving breadth were written differently, with each question presented in Chinese only and all the options for each question presented in Vietnamese only to examine whether participants knew the equivalent Vietnamese meaning of the Chinese word; this approach drew on experience from a previous study (Nation and Beglar, 2007). The two authors who were native speakers of Chinese were responsible for proofreading and revising the Chinese version of the questionnaires, and a native Vietnamese teacher from the university who had majored in the Chinese language was invited to translate and edit the Vietnamese version. The two questionnaires were distributed to the participants by a Chinese language teacher from the university in Vietnam *via* an online format, namely, Google Forms, and were required to be completed during the class with a maximum duration of 35 min.

#### Questionnaire 1: Self-Regulating Capacity in Vocabulary Learning

This questionnaire was designed to investigate the participants’ self-regulation performance in L2 vocabulary learning. The questionnaire was derived from the self-regulation capacity in vocabulary learning instrument by Tseng et al. (2006), which includes commitment control, metacognitive control, satiation control, emotion control, and environmental control, with 20 items in total. The commitment control items indicate the retention and growth of the learner’s learning goals (i.e., *When learning vocabulary, I believe I can achieve my goals more quickly than expected*). The metacognitive control items concern the learner’s monitoring and control of his or her attention (i.e., *When learning vocabulary, I have special techniques to keep my concentration focused).* The satiation control items display increased interest in learning for learners (i.e., *During the process of learning vocabulary, I feel satisfied with the ways I eliminate boredom*). The emotion control items concern the management of personal emotions (i.e., *I feel satisfied with the methods I use to reduce the stress of vocabulary learning*). The environmental control items indicate the influence of the external environment on the learner (i.e., *When I study vocabulary, I look for a good learning environment*).

As the study aimed to clarify the relationship between self-regulation and vocabulary knowledge (depth and breadth) rather than investigate Vietnamese university students’ Chinese learning, it was necessary to determine the internal factor structure of self-regulation. To ensure that the questionnaire could reflect the *status quo* of the participants, based on the reliability and validity analysis of the two questionnaires, this study adopted exploratory factor analysis (EFA) to extract potential factors and excluded items that did not fit the category. In addition, confirmatory factor analysis (CFA) was used to verify the results of EFA and determine the factor structure within self-regulation.

The reliability of the questionnaire was analyzed by SPSS 27, and the Cronbach’s α = 0.965 > 0.9. In addition, a factor analysis of the questionnaire was conducted to suggest questions with correlations. The Kaiser–Meyer–Olkin (KMO) test and Bartlett’s test of sphericity were used with the result that KMO = 0.946 and *p* < 0.001, which signaled a high level of significance (Kaiser, 1974). The data derived from the questionnaire were then subjected to EFA and CFA, for which both SPSS 27 and AMOS 27 were used. The results are shown in Tables 1, 2.

**TABLE 1:** Factors extracted from exploratory factor analysis (EFA).

Items	Number	Emotion control	Metacognitive control
Self-regulating capacity in vocabulary learning	2	0.648	
	3	665	
	4	0.765	
	5	0.861	
	6	0.781	
	19	0.764	
	10		0.695
	11		0.831
	12		0.74
	14		0.809
	16		0.777
	Variance explained in percentage Total 67.062%	60.685%	6.377%
	Cronbach’s α Total 932	0.907	0.9

**TABLE 2:** Commonly used confirmatory factor analysis (CFA) fit indices.

	CMIN/DF	GFI	CFI	TLI	IFI	NFI	RMSEA
Acceptable level	<3	>0.9	>0.9	>0.9	>0.9	>0.9	≤0.08
Self-regulating capacity in vocabulary learning	2.242	0.923	0.968	0.966	0.969	0.945	0.078

First, EFA extracted two factors, which were renamed based on previous research (Tseng et al., 2006), and the items with low factor loadings (<0.6) were removed. The names of the factors were emotion control and metacognitive control. The former indicates the learner’s capability to regulate and manage negative emotions to induce positive personal feelings (items 2, 3, 4, 5, 6, 19). The latter indicates the learner’s monitoring and control of attention to reduce unnecessary delays in the learning process (items 10, 11, 12, 14, 16). These two factors could cumulatively explain 67.062% of the variance. Second, based on the CFA results (see Table 2), all requirements were met. Both the GFI and CFI had values >0.9, and the RMSEA was.078, thereby meeting the criterion of less than 0.08 (Bryant and Yarnold, 1995). The results of the validation factor analysis indicate that the model is stable and reliable and can be subjected to subsequent analysis.

#### Questionnaire 2: Vocabulary Knowledge (Breadth and Depth)

This questionnaire consists of two parts: vocabulary breadth and depth. The items concerning breadth were adapted from “The Vocabulary Size Test” (Nation and Beglar, 2007; Thi and Nation, 2011) to investigate the participants’ understanding and knowledge of the target vocabulary, while the items regarding depth were adapted from the Word Associates Test (Nation, 1986) to examine the extent to which the participants understood the form and meaning of the target vocabulary. The Chinese vocabulary was selected by referring to the “Frequency-based HSK Vocabulary” (Yang, 2016). The maximum score is 100 for the items on depth and breadth, while the minimum score is 0 for these items.

A total of fifty words were selected randomly from the superhigh frequency vocabulary lists provided in HSK 2–6 and were used to form fifty items concerning breadth, with each item valuing 2 points for a correct answer. If the participant failed to obtain the correct answer, no points were given. The reliability of breadth was 0.791, which reached the acceptable level of reliability according to previous studies (Alderson et al., 1995). With reference to the study on L2 learners’ vocabulary testing (Nation and Beglar, 2007), all questions on vocabulary breadth were presented in Chinese, and the options were presented in Vietnamese. One of the reasons for choosing such a bilingual method is that the bilingual testing approach is a proven method for examining students’ understanding of target words (Nation and Beglar, 2007; Nation, 2011). Another reason is that such a method facilitates Vietnamese university students to complete the items efficiently without the intervention of external factors. Excerpt 1 is an example of one item on breadth, which requires the participants to select the correct response out of four options based on the prompted statements. Participants were required to choose the equivalent meaning of *现在* from the four Vietnamese words; (b) *Bây giờ* is the correct answer.

*Excerpt 1*

1. 现在：你现在去机场
(a) Phát hiện
(b) Bây giờ
(c) Đang
(d) Là

The same fifty words that were used for the items on breadth were employed to construct 100 items, with two items for each word for the items concerning vocabulary depth and one point was given for the correct answer to each item. Points were given to correct answers only. The reliability for the depth was 0.877, which was also an acceptable level of reliability (Alderson et al., 1995). Based on previous work (Nation, 1986), all the questions and options in this questionnaire were expressed in both Chinese and Vietnamese. Excerpt 2 is an example of two items on depth involving the same word. The first item requires the participants to select the synonym or antonym of the given word. The second question asks the participants to choose a word that could form a meaningful phrase or sentence with the given word.

*Excerpt 2*

1. 现在Bây giờ
(a) 衣服 quần áo
(b) 香蕉 chuối
(c) 以前 trước đây
(d) 校长 hiệu trưởng
2. 现在Bây giờ
(a) 去 đi
(b) 次 lần
(c) 二 hai
(d) 个 cái

Option (c)*以前 trước đây* is the correct answer for the first item because it is the antonym of the Chinese word *现在Bây giờ.* Option (b) *去 đi* is the correct choice for the second item as it can be combined with *现在Bây giờ* to form the sentence *现在去机场*.

### Procedure

First, in this study, the data were collected by the two authors with the help of a Chinese language teacher from a university in Vietnam (hereinafter referred to as “the teacher”). The two authors of this study were responsible for designing the questionnaires and processing and analyzing the data, while the teacher was responsible for the translation and distribution of the questionnaires. The two authors and the teacher contributed to both the pilot study and the final data collection. In the earliest recruitment phase, the teacher helped in post-recruitment information and contacted the participants by sending them an electronic consent form. In the electronic consent form, the purpose and method of this study were introduced in detail, including information related to the confidentiality, anonymity, storage, and use of data. It was stated that the data collected in this study would only be used for research purposes. The teacher invited all the participants who sent back their signed consent forms into a classroom to complete the two questionnaires through Google Forms. Information regarding participation, such as time and classroom number, was provided to the participants by the teacher when the participants signed up to participate. Participants were asked to bring mobile electronic devices such as cell phones or laptops to complete the two online questionnaires.

Second, the teacher and authors explained the content of the questionnaires and the related guidelines before the study began. As the online method was adopted for data collection, participants were allowed to use electronic devices such as cell phones, laptops, or tablets in this study. However, their use was limited to completing the questionnaires. No searching for the meaning of the concerned Chinese words or exchanging answers with others was allowed. A pilot study was conducted before the questionnaire was administered. The purpose of the pilot study was to check for possible problems in questionnaire administration and ensure that the formal data collection could be smoothly conducted. The pilot study was performed from 8 to 14 June 2021 with 35 participants (23 females and 12 males). A total of 20 min was given to complete the two questionnaires. However, the results of the pilot study showed that the participants could not finish both questionnaires in the given time. Although most of them completed the survey in approximately 30 min, after considering the variances in the speed of over 200 participants, a maximum duration of 35 min was given to each participant. During the period of formal data collection, the teacher stayed in the classroom and kept in contact with the first author *via* zoom throughout the whole process to monitor the participants and provide prompt assistance if any item confused them during the survey. The questionnaires were distributed to the participants *via* a Google Form link. A maximum of 35 min was given to complete the two questionnaires, and any overdue submissions were not accepted.

When the participants finished the items related to personal information, such as gender and study time, their interface would automatically jump to Q1 and Q2 would appear upon the completion of Q1. The participants were required to submit the questionnaires and tell the teacher or the first author immediately once they finished. Participants who had completed the task could choose to leave the classroom or wait until the end of the survey. If they decided to wait until the end, they could not use electronic devices or communicate with others. The results of the vocabulary knowledge questionnaire were described in terms of the mean, standard deviation, skewness, and kurtosis. Subsequently, a structural equation model was developed *via* using AMOS 27. The interactions between the self-regulation factors were discussed. Additionally, the predicted role of self-regulation in vocabulary depth and breadth was subsequently analyzed.

## Results

### Descriptive Statistics (Vocabulary Knowledge)

**FIGURE 1:**
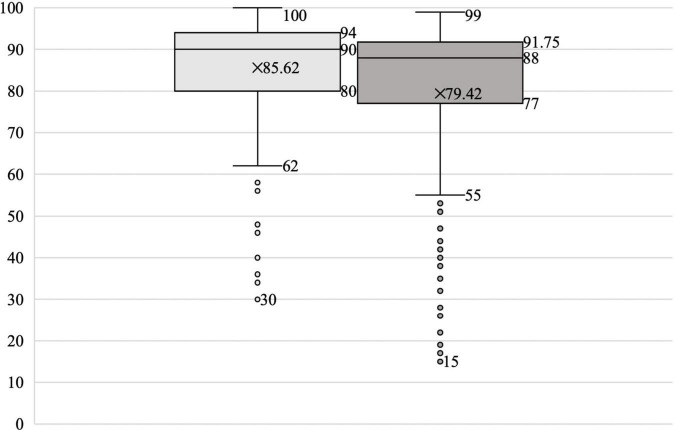
Vocabulary knowledge (breadth and depth).

A total of 215 Vietnamese university students completed the vocabulary knowledge questionnaire. For the items concerning vocabulary breadth, the minimum and maximum values were 30 and 100, respectively (*M* = 85.62, *SD* = 12.73) (see Table 3 and Figure 1). For the items about vocabulary depth, the minimum value was 15, and the maximum value was 100 (*M* = 79.42, *SD* = 19.78).

**TABLE 3:** Statistics.

		Breadth	Depth
*N*	Valid	204	204
	Missing	0	0
Mean		85.62	79.42
Std. error of mean		0.89	1.38
Media		90	88
Std. deviation		12.73	19.78
Variance		162	391.06
Minimum		30	15
Maximum		100	99
Skewness		−1.99	−1.6
Kurtosis		4.28	1.52

The results in Table 3 show that the mean values for vocabulary breadth and depth were high. However, vocabulary breadth was higher than vocabulary depth regarding the minimum values. The values of the standard deviation and variance for vocabulary breadth were also smaller than those for vocabulary depth. Both the breadth and depth values were smaller than 0 in terms of skewness. The absolute value of breadth was larger than that of depth, and both were negatively skewed. Both the kurtosis values were greater than 0. The value for depth was 4.28 > 3, which was leptokurtic. The breadth value was 1.52 < 3, which was platykurtic.

Based on the data in the box plot (see Figure 1), the interquartile range for breadth and depth was 14 and 14.75, respectively. The breadth was less than the depth. However, at the low and upper quartiles, the breadth was 80 and 94, and the depth was 77 and 91.75. The breadth results were higher than the depth results. Moreover, the outliers were on the lower side for both breadth and depth. The number of outliers for breadth was smaller, and there were significantly more outliers for depth than breadth, with multiple extreme outliers. However, a few students had poor scores in depth.

In general, Vietnamese university students showed differences in their performance regarding vocabulary knowledge. The total results for breadth were positive, with concentrated scores and small gaps between students. The results for depth were more widely distributed, with significant variances between students.

### Self-Regulation Components

**FIGURE 2:**
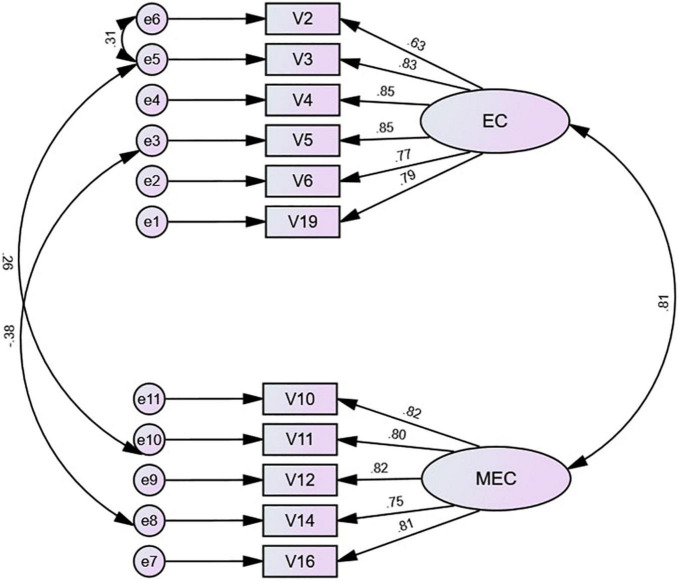
The structural equation model of self-regulation. EC, emotion control items, MEC, metacognitive control items.

Based on the results of EFA and CFA, we identified the existence of two factors of self-regulation among Vietnamese university students, namely, emotion control and metacognitive control (see Table 1). The former is mainly concerned with regulating negative emotions, and the latter is concerned with attention regulation and control (Corno and Kanfer, 1993; Dörnyei, 2001; Tseng et al., 2006). The two factors cumulatively explain 67.062% of the variance with high explanatory power. Subsequently, we used AMOS 27 to set a structural equation model and explore the structural relationships between the factors inherent in self-regulation (see Figure 2).

The standardized path coefficient between emotion control and metacognitive control was 0.81 (*p* < 0.05; it reached a significant level and was positively correlated) (Bryant and Yarnold, 1995). Thus, an increase in emotion control leads to improvements in the metacognitive control level and ultimately to enhancements of self-regulation. On the one hand, this result reflects a high positive correlation between the internal factors of self-regulation, and the two promote each other. On the other hand, it also indicates that Vietnamese university students regulate and manage their negative emotions in Chinese vocabulary learning and pay attention to reducing unnecessary delays in the learning process.

### Contributions of Self-Regulation to Vocabulary Knowledge

After analyzing the results of vocabulary knowledge and the internal factor structure of self-regulation, we utilized AMOS 27 to build structural equation models to discuss the relationship between self-regulation and vocabulary knowledge (depth and breadth). After various adjustments, the results indicate acceptable fit indices (CMIN/DF = 1.683, GFI = 0.926, CFI = 0.975, TLI = 0.976, IFI = 0.975, NFI = 0.94, RMSEA = 0.058), indicating that the model was stable and reliable. Further path analysis is shown in Table 4.

**TABLE 4:** Path coefficients of self-regulation and vocabulary knowledge.

	β	S.E.	C.R.	*P*
**Total effects**				
Metacognitive control–breadth	–0.188	1.285	–2.584	0.01
Metacognitive control–depth	–0.101	1.731	0.886	< 0.001
Breadth–depth	0.537	0.902	9.081	< 0.001
**Direct effects**				
Metacognitive control–breadth	–0.188	1.285	–2.584	< 0.001
Breadth–depth	0.537	0.902	9.081	< 0.001
**Indirect effects**				
Metacognitive control–depth	–0.101	1.731	0.886	< 0.001

**FIGURE 3:**
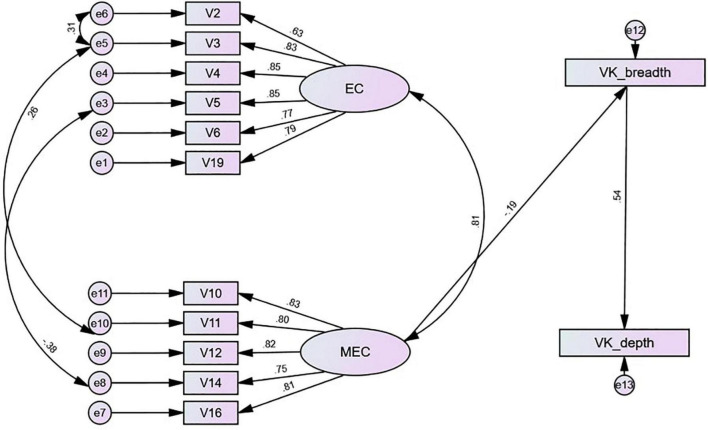
Self-regulation and vocabulary knowledge. EC, emotion control items, MEC, metacognitive control items.

Based on the analysis in Table 4, self-regulation directly affected vocabulary breadth, with both path coefficients reaching significance levels (*p* < 0.05). There was a direct effect between metacognitive control and vocabulary breadth. The standardized path coefficient was -0.19 and *p* < 0.001 (see Table 4 and Figure 3). Metacognitive control also affected vocabulary depth indirectly through vocabulary breadth. The indirect effect was -0.101 and *p* < 0.001, indicating a high negative correlation between metacognitive control and vocabulary knowledge. As metacognitive control decreased, the result of Vietnamese university students’ vocabulary depth and breadth questionnaire significantly increased. The same direct effect existed for vocabulary knowledge (breadth and depth). The standardized path coefficient was 0.537, *p* < 0.001, demonstrating a positive correlation and indicating that Vietnamese university students who presented excellent results in vocabulary breadth would also obtain better scores in depth. Moreover, there was no effect between emotion control and vocabulary knowledge (depth and breadth) (see Figure 3). Overall, self-regulation had a predictive effect on vocabulary knowledge, but this effect was negative.

## Discussion

### Analysis of the Vocabulary Knowledge (Breadth and Depth) Results

Most Vietnamese university students could read, write, listen, and express themselves in Chinese after 3 or 4 years of systematic Chinese language study. These abilities helped them obtain a high score on the breadth of vocabulary knowledge. The results in Figure 1 demonstrate that most of the participants obtained high scores in breadth, with a relatively stable overall result. However, the depth score varied in different participants with many outliers. It could be assumed that most Vietnamese university students could identify the vocabulary in the vocabulary knowledge questionnaire. However, they were challenged by the form and meaning of the vocabulary. Identifying synonyms and antonyms and constructing sentences with given words are still challenging for some Vietnamese university students, which indicates that semantic understanding and identification of multiple word forms are still inadequate for those L2 learners. This finding was particularly notable among the participants with low Chinese language capability. After 3 or 4 years of Chinese vocabulary learning, most Vietnamese university students have a high Chinese vocabulary level. They can understand the basic meaning of Chinese words, which meets their basic needs for Chinese learning, such as reading in Chinese and communicating with Chinese speakers daily. However, even after completing the *Chinese vocabulary course*, most Vietnamese university students are still unable to understand, identify, and flexibly use sufficient Chinese words precisely. This result reflects the significant difference between depth and vocabulary breadth and reveals that Vietnamese university students have defects in the form, meaning, and usage of Chinese words.

The purpose of vocabulary knowledge classification is to detect L2 learners’ vocabulary level, clarify the relationship between various vocabulary components, and improve L2 vocabulary learning effectiveness (Stæhr, 2009; Webb, 2013; Zhang and Lu, 2015; Luo et al., 2021; Teng, 2021). The words selected in this study are high-frequency words from HSK 2–6. Vocabulary breadth was used to investigate the participants’ understanding of vocabulary, while depth was used to examine their comprehension of the form and meaning of the vocabulary. Differences were found in vocabulary depth and breadth among Vietnamese university students, which indicates that Vietnamese university students successfully grasp some significant aspects of the word meaning of Chinese words but not the form, usage, or extensional meaning. Although the premise of mastering the form, usage, and extensional meaning of Chinese words is to understand significant aspects of word meaning correctly, most Vietnamese university students still fail to break through the surface meaning to grasp the profound meaning of Chinese words after a period of learning. In addition, depth includes breadth and constitutes two levels of vocabulary knowledge together with breadth, both of which are important indicators to measure the effectiveness of L2 learning (Qian and Lin, 2020; Yanagisawa and Webb, 2020). In other words, this study concluded that items in breadth represented the size of the participants’ Chinese vocabulary, and items in depth indicated their actual Chinese language level and capability. Furthermore, according to the results of the structural equation model, this study concluded that Vietnamese university students can regulate their emotions and that their self-regulation capability is significantly correlated with their learning monitoring. For example, regulating personal emotions through self-encouragement and relaxation helps promote interest in learning (Corno and Kanfer, 1993; Dörnyei, 2001). Various forms of Chinese learning activities, such as online writing exercises, presentations, and group presentations, are conducive to learners’ strong interest in Chinese learning (Pham et al., 2020). It is also necessary to stress maintaining monitoring skills. Teachers can guide students to establish milestones in Chinese vocabulary learning and help them make progress step by step, which benefits students to move on to the next level of learning based on their current knowledge.

### Relationship Between Self-Regulation Variables and Vocabulary Knowledge (Breadth and Depth)

This study confirmed a correlation between emotion control and metacognitive control. Emotion control involves learners’ control and management of negative emotions and the generation of positive personal emotions, while metacognitive control concerns learners’ monitoring of personal attention and thus reduces unnecessary distractions and delays (Corno and Kanfer, 1993; Dörnyei, 2001; Tseng et al., 2006). The self-regulation of 204 Vietnamese university students who participated in this study was mainly manifested in the regulation of personal emotions and monitoring of attention. Therefore, Vietnamese university students can improve their self-confidence by maintaining their interest in Chinese vocabulary learning. They can reduce the emergence of negative emotions and maintain high motivation for Chinese language learning. For example, they can fully understand the sources of Chinese vocabulary before class and strengthen their practice of target vocabulary after class to ensure that the factors that may interfere with and affect the vocabulary learning process are minimized. However, this result also reflects that emotion and attention play essential roles in vocabulary learning for Vietnamese university students. Once there is a significant fluctuation between the two, such as changes in personal emotion and attention, Chinese vocabulary learning in Vietnamese universities will be directly or indirectly affected. More importantly, both emotion control and metacognitive control involve learners’ regulation of negative states (Tseng et al., 2006). Therefore, attention needs to be attached to the changes in Vietnamese university students’ emotional regulation and attention.

The analysis results also show that emotion control in self-regulation did not affect vocabulary knowledge (depth and breadth). The monitoring of individual attention is often considered the first step in language learning (Tseng et al., 2006). The participants in this study had a certain number of years of experience in Chinese language learning and could complete the vocabulary depth and breadth questionnaire independently within a specified time frame. In addition, there was a direct negative relationship between metacognitive control and vocabulary breadth. Metacognitive control also affected depth indirectly through breadth, which indicates that the emotional regulation and management of Vietnamese university students directly affect their understanding of the significant aspects of word meaning and indirectly affect their mastery of the form, meaning, and usage of words. However, since it is a negative correlation, the capability of Vietnamese university students to monitor their negative emotions may facilitate their vocabulary learning process to a certain extent. Nevertheless, excessive monitoring may have a negative effect. Vietnamese university students were concerned with monitoring and managing personal attention in the process of self-regulation to reduce procrastination or inefficient vocabulary learning. Such regulation does not eventually affect the learning of vocabulary knowledge (depth and breadth). There is a negative correlation between emotion control and vocabulary breadth, and most Vietnamese university students obtained high scores in vocabulary breadth. Although Vietnamese university students attentionally eliminate negative emotions during vocabulary learning, the capability of Vietnamese university students to monitor their negative emotions may facilitate their vocabulary learning to a certain extent.

As an essential theory in educational psychology, self-regulation has a vital role in enhancing L2 learning (Zimmerman and Risemberg, 1997). The SRCs of Vietnamese university students are mainly manifested in the regulation of negative personal emotions and the supervision of attention in Chinese vocabulary learning. This study found that excessive emotional regulation affects learners’ vocabulary knowledge. In particular, there is a significant decline in breadth. Although depth is not directly affected by self-regulation, the indirect effect of emotion regulation deserves the attention of researchers. It is necessary to maintain a stable level of self-regulation to help improve the effectiveness of Chinese vocabulary learning. Conversely, excessive emotional regulation can impair learners’ vocabulary knowledge, especially at the vocabulary level. Furthermore, it even affects Vietnamese university students’ L2 vocabulary learning progress and decreases L2 learning effectiveness.

Therefore, it is recommended that teachers reduce excessive after-school exercises and assignments and retain the right amount of Chinese learning tasks, which will allow Vietnamese university students to have sufficient time to understand the content and flow of the current scheme, promptly regulate their negative personal emotions, and promote their interest in learning Chinese. Teachers can also increase independent learning time by providing more free online learning resources. Vietnamese university students should be provided with sufficient time to regulate their negative emotions to ensure that their SRCs are excellent.

## Conclusion

This study concluded that the results of vocabulary breadth were better than those of vocabulary depth. Vietnamese university students of different levels showed a significant gap in the results of vocabulary depth. Subsequent factor analyses revealed two self-regulation factors among Vietnamese university students. Metacognitive control directly affected breadth, with a negative correlation, and depth had an indirect effect through breadth. However, there was no predictive effect between depth and self-regulation. For such a result, we believe that the current results of vocabulary breadth represent only the participants’ recognition of Chinese vocabulary. In contrast, the result of the vocabulary depth indicates their actual Chinese language level and capability. The participants’ motivation allowed them to have better SRCs. However, excessive emotional regulation can cause a decline in personal vocabulary knowledge. Theoretically, this study builds on previous work to emphasize that appropriate self-regulation promotes vocabulary knowledge levels. On this basis, it is suggested that teachers of Chinese in Vietnamese universities should pay attention to the role of negative SRCs in vocabulary knowledge and maintain students’ SRCs in a relatively stable state.

There are also some limitations to this study. This study quantitatively examined Vietnamese university students’ Chinese vocabulary knowledge, thereby making it impossible to measure such students’ Chinese vocabulary learning from a long-term perspective. When Vietnamese university students learn Chinese vocabulary, are there other factors that affect their vocabulary knowledge (breadth and depth) apart from self-regulation? This issue needs further investigation.

## Data Availability Statement

The raw data supporting the conclusions of this article will be made available by the authors, without undue reservation.

## Ethics Statement

The studies involving human participants were reviewed and approved by Human Research Ethics Program at The Education University of Hong Kong. Written informed consent for participation was not required for this study in accordance with the national legislation and the institutional requirements.

## Author Contributions

CW and SZ: substantial contributions to the conception or design of the work, research design, and implementation. SZ: data analysis, processing, and drafting of the work. CW: revising it critically for important intellectual content. Both authors contributed to the article and approved the submitted version.

## Conflict of Interest

The authors declare that the research was conducted in the absence of any commercial or financial relationships that could be construed as a potential conflict of interest.

## Publisher’s Note

All claims expressed in this article are solely those of the authors and do not necessarily represent those of their affiliated organizations, or those of the publisher, the editors and the reviewers. Any product that may be evaluated in this article, or claim that may be made by its manufacturer, is not guaranteed or endorsed by the publisher.

## References

[B1] Abdolrezapour P. Ghanbari N. (2021). Enhancing learning potential score in EFL listening comprehension and self-regulation through self-regulated dynamic assessment procedures. *Lang. Test Asia* 11:10. 10.1186/s40468-021-00126-5

[B2] Alderson J. C. Clapham C. Wall D. (1995). *Language Test Construction and Evaluation.* Cambridge: Cambridge University Press.

[B3] Andrade M. S. Evans N. W. (2012). *Principles and Practices for Response in Second Language Writing: Developing Self-Regulated Learners*, 1st ed. England: Routledge.

[B4] August D. Shanahan T. Escamilla K. (2009). English language learners: developing literacy in second language learners—Report of the national literacy panel on language-minority children and youth. *J. Literat. Res.* 41 432–452. 10.1080/10862960903340165

[B5] Birgisdottir F. Gestsdottir S. Geldhof G. J. (2020). Early predictors of first and fourth grade reading and math: the role of self-regulation and early literacy skills. *Early Child. Res. Quart.* 53 507–519. 10.1016/j.ecresq.2020.05.001

[B6] Bodrova E. Leong D. J. (2007). *Tools of the Mind: The Vygotskian Approach to Early Childhood Education*, 2nd Edn. Columbus: Merrill/Prentice Hall.

[B7] Bohlmann N. L. Downer J. T. (2016). Self-regulation and task engagement as predictors of emergent language and literacy skills. *Early Educ. Dev.* 27 18–37. 10.1080/10409289.2015.1046784

[B8] Bohlmann N. L. Maier M. F. Palacios N. (2015). Bidirectionality in self-regulation and expressive vocabulary: comparisons between monolingual and dual language learners in preschool. *Child Dev.* 86 1094–1111. 10.1111/cdev.12375 25906925

[B9] Bryant F. B. Yarnold P. R. (1995). “Principal-components analysis and exploratory and confirmatory factor analysis,” in *Reading and Understanding Multivariate Statistics*, eds Grimm L. G. Yarnold P. R. (Washington: American Psychological Association), 99–136.

[B10] Cadima J. Barros S. Ferreira T. Serra-Lemos M. Leal T. Verschueren K. (2019). Bidirectional associations between vocabulary and self-regulation in preschool and their interplay with teacher-child closeness and autonomy support. *Early Child. Res. Quart.* 46 75–86. 8.04.004 10.1016/j.ecresq.201

[B11] Chen C. Truscott J. (2010). The effects of repetition and L1 lexicalization on incidental vocabulary acquisition. *Appl. Linguist.* 31 693–713. 10.1093/applin/amq031

[B12] Connor C. M. Day S. L. Phillips B. Sparapani N. Ingebrand S. W. McLean L. (2016). Reciprocal effects of self-regulation, semantic knowledge, and reading comprehension in early elementary school. *Child Dev.* 87 1813–1824. 10.1111/cdev.12570 27264645 PMC5138137

[B13] Corno L. Kanfer R. (1993). The role of volition in learning and performance. *Rev. Res. Educ.* 19 301–341. 10.2307/1167345

[B14] DiBenedetto M. K. (2018). “Self-regulation in secondary classrooms: Theoretical and research applications to learning and performance,” in *Connecting Self-Regulated Learning and Performance with Instruction Across High School Content Areas*, ed. DiBenedetto M. K. (Cham: Springer), 3–23. 10.1007/978-3-319-90928-8_1

[B15] Dörnyei Z. (2001). *Motivational Strategies in the Language Classroom.* Cambridge: Cambridge University Press, 10.1017/CBO9780511667343

[B16] Dörnyei Z. (2005). “Language learning strategies and student self-regulation,” in *The Psychology of the Language Learner: Individual differences in Second Language Acquisition*, ed. Dörnyei Z. (England: Routledge), 162–196.

[B17] Fan N. (2020). Strategy use in second language vocabulary learning and its relationships with the breadth and depth of vocabulary knowledge: a structural equation modeling study. *Front. Psychol.* 11:752. 10.3389/fpsyg.2020.00752 32477204 PMC7237738

[B18] Fischer K. W. Bidell T. R. (2006). “Dynamic development of action and thought,” in *Handbook of Child Psychology: Theoretical models of Human Development*, eds Lerner R. M. Damon W. (Hoboken: John Wiley & Sons Inc), 313–399.

[B19] Fuhs M. W. Day J. D. (2011). Verbal ability and executive functioning development in preschoolers at head start. *Dev. Psychol.* 47 404–416. 10.1037/a0021065 21142363

[B20] Fuhs M. W. Nesbitt K. T. Farran D. C. Dong N. (2014). Longitudinal associations between executive functioning and academic skills across content areas. *Dev. Psychol.* 50 1698–1709. 10.1037/a0036633 24749550

[B21] Fung W. K. Chung K. K. H. Cheng R. W. Y. (2019). Gender differences in social mastery motivation and its relationships to vocabulary knowledge, behavioral self-regulation, and socioemotional skills. *Early Educ. Dev.* 30 280–293. 10.1080/10409289.2018.1544004

[B22] González-Fernández B. (2022). Conceptualizing L2 Vocabulary Knowledge: An Empirical Examination of The Dimensionality of Word Knowledge. *Stud. Sec. Lang. Acqu.* 1–31. 10.1017/S0272263121000930

[B23] González-fernández B. Schmitt N. (2020). Word knowledge: exploring the relationships and order of acquisition of vocabulary knowledge components. *Appl. Linguist.* 41 481–505. 10.1093/applin/amy057

[B24] Gunning P. Oxford R. L. (2014). Children’s learning strategy use and the effects of strategy instruction on success in learning ESL in Canada. *System* 43 82–100. 10.1016/j.system.2013.12.012

[B25] Hromalik C. D. Koszalka T. A. (2018). Self-regulation of the use of digital resources in an online language learning course improves learning outcomes. *Dist. Educ.* 39 528–547. 10.1080/01587919.2018.1520044

[B26] Kaiser H. F. (1974). An index of factorial simplicity. *Psychometrika* 39 31–36. 10.1007/BF02291575

[B27] Kim D. H. Wang C. Truong T. N. N. (2021). Psychometric properties of a self-efficacy scale for English language learners in Vietnam. *Lang. Teach. Res.* 1–16. 10.1177/13621688211027852

[B28] Lan P. S. Liu M. C. Baranwal D. (2020). Applying contracts and online communities to promote student self-regulation in English learning at the primary-school level. *Interact. Learn. Environ.* 28, 1–12. 10.1080/1049482

[B29] Luo Y. Song H. Wan L. Zhang X. (2021). The effect of vocabulary depth and breadth on English listening comprehension can depend on how comprehension is neasured. *Front. Psychol.* 12:657573. 10.3389/fpsyg.2021.657573 34113291 PMC8185333

[B30] Ma R. Oxford R. L. (2014). A diary study focusing on listening and speaking: the evolving interaction of learning styles and learning strategies in a motivated, advanced ESL learner. *System* 43 101–113. 10.1016/j.system.2013.12.010

[B31] Manchón R. M. (2020). *Writing and Language Learning: Advancing Research Agendas.* Amsterdam: John Benjamins.

[B32] Mathis E. T. B. Bierman K. L. (2015). Dimensions of parenting associated with child prekindergarten emotion regulation and attention control in low-income families. *Soc. Dev.* 24 601–620. 10.1111/sode.12112 26195853 PMC4505750

[B33] Matthews J. (2018). Vocabulary for listening: emerging evidence for high and mid-frequency vocabulary knowledge. *System* 72 23–36. 7.10.005 10.1016/j.system.201

[B34] McClelland M. M. Cameron C. E. Connor C. M. Farris C. L. Jewkes A. M. Morrison F. J. (2007). Links between behavioral regulation and preschoolers’ literacy, vocabulary, and math skills. *Dev. Psychol.* 43 947–959. 10.1037/0012-1649.43.4.947 17605527

[B35] Melka F. (1997). “Receptive versus productive aspects of vocabulary,” in *Vocabulary: Description, Acquisition, and Pedagogy*, eds Schmitt N. McCarthy M. (Cambridge: Cambridge University Press), 84–102. 10.1016/j.ehb.2017.02.002

[B36] Morrison F. J. Ponitz C. C. McClelland M. M. (2010). “Self-regulation and academic achievement in the transition to school,” in *Child Development at the Intersection of Emotion and Cognition*, eds Calkins S. D. Bell M. A. (Washington, D.C: American Psychological Association), 203–224. 10.1037/12059-011

[B37] Naderifar A. (2018). The comparative effect of concept mapping and vocabulary notebook keeping on Iranian EFL learners’ self-regulation in vocabulary learning. *Cogent Educ.* 5:1491782. 10.1080/2331186X.2018.1491782

[B38] Nation I. S. P. (1983). Testing and teaching vocabulary. *Guidelines* 5 12–25.

[B39] Nation I. S. P. (1986). *Vocabulary lists: Words, Affixes and Stems.* New Zealand: English Language Institute, Victoria University of Wellington.

[B40] Nation I. S. P. (2011). Research into practice: Vocabulary. *Lang. Teach.* 44 529–539. 10.1017/S0261444811000267

[B41] Nation I. S. P. (2020). “The different aspects of vocabulary knowledge,” in *The Routledge Handbook of Vocabulary Studies*, ed. Webb S. (England: Routledge), 15–29. 10.4324/9780429291586-2

[B42] Nation I. S. P. Beglar D. (2007). A vocabulary size test. *Lang. Teach.* 31 9–13.

[B43] Nietfeld J. L. Shores L. R. Hoffmann K. F. (2014). Self-regulation and gender within a game-based learning environment. *J. Educ. Psychol.* 106 961–973. 10.1037/a0037116

[B44] Oxford R. L. (2011). *Teaching and Researching: Language Learning Strategies.* England: Routledge.

[B45] Pham T. H. T. Zhang Y. C. Shang J. (2020). Students of Chinese language major and their attitude towards the current learning study in Vietnam. *Front. Educ. Technol.* 3:p12. 10.22158/fet.v3n1p12

[B46] Phan N. T. T. Locke T. (2015). Sources of self-efficacy of Vietnamese EFL teachers: a qualitative study. *Teach. Teach. Educ.* 52 73–82. 10.1016/j.tate.2015.09.006

[B47] Phuong H. Y. (2017). Task-based language teaching and its impact on Vietnamese students’ use of self-regulated learning strategies in a writing classroom. *Can Tho Univ. J. Sci.* 5 30–38. 10.22144/ctu.jen.2017.004

[B48] Puimège E. Peters E. (2019). Learners’ English vocabulary knowledge prior to formal instruction: the role of learner-related and word-related variables. *Lang. Learn.* 69 943–977. 10.1111/lang.12364

[B49] Qian D. D. Lin L. H. F. (2020). “The relationship between vocabulary knowledge and language proficiency,” in *The Routledge Handbook of Vocabulary Studies*, ed. Webb S. (England: Routledge), 66–80. 10.4324/9780429291586-5

[B50] Ramsook K. A. Welsh J. A. Bierman K. L. (2020). What you say, and how you say it: Preschoolers’ growth in vocabulary and communication skills differentially predict kindergarten academic achievement and self-regulation. *Rev. Soc. Dev.* 29 783–800. 10.1111/sode.12425 33041538 PMC7546440

[B51] Rasekh Z. E. Ranjbary R. (2003). Metacognitive strategy training for vocabulary learning. *Teach. Eng. Sec. Foreign Lang.* 7:15.

[B52] Raver C. C. Jones S. M. Li-Grining C. Zhai F. Bub K. Pressler E. (2011). CSRP’s impact on low-income preschoolers’ preacademic skills: self-regulation as a mediating mechanism. *Child Dev.* 82 362–378. 7-8624.2010.01561.x 10.1111/j.146 21291447 PMC3682645

[B53] Schmitt N. (2010). *Researching Vocabulary: A Vocabulary Research Manual.* London: Palgrave Macmillan.

[B54] Schmitt N. (2014). Size and depth of vocabulary knowledge: what the research shows. *Lang. Learn.* 64 913–951. 10.1111/lang.12077

[B55] Schmitt N. Schmitt D. Clapham C. (2001). Developing and exploring the behaviour of two new versions of the vocabulary levels test. *Lang. Test* 18 55–88. 10.1177/026553220101800103

[B56] Seker M. (2015). The use of self-regulation strategies by foreign language learners and its role in language achievement. *Lang. Teach. Res.* 20 600–618. 10.1177/1362168815578550

[B57] Stæhr L. (2009). Vocabulary knowledge and advanced listening comprehension in English as a foreign language. *Stud. Second Lang. Acquisit.* 31, 577–607. 10.1017/S0272263109990039

[B58] Stoeger H. Sontag C. Ziegler A. (2014). Impact of a teacher-led intervention on preference for self-regulated learning, finding main ideas in expository texts, and reading comprehension. *J. Educ. Psychol.* 106 799–814. 10.1037/a0036035

[B59] Su Y. Li Y. Liang J. C. Tsai C. C. (2019). Moving literature circles into wiki-based environment: the role of online self-regulation in EFL learners’ attitude toward collaborative learning. *Comp. Assist. Lang. Learn.* 32 556–586. 10.1080/09588221.2018.1527363

[B60] ten Braak D. Størksen I. Idsoe T. McClelland M. (2019). Bidirectionality in self-regulation and academic skills in play-based early childhood education. *J. Appl. Dev. Psychol.* 65:101064. 10.1016/j.appdev.2019.101064

[B61] Teng M. F. (2021). Exploring awareness of metacognitive knowledge and acquisition of vocabulary knowledge in primary grades: a latent growth curve modelling approach. *Lang. Aware.* 1–25. 10.1080/09658416.2021.1972116

[B62] Thi L. Nation P. (2011). A bilingual vocabulary size test of English for Vietnamese learners. *RELC J.* 42, 86–99. 10.1177/0033688210390264

[B63] Tian L. Liu Q. Zhang X. (2022). Self-regulated writing strategy use when revising upon automated, peer, and teacher feedback in an online English as a foreign language writing course. *Front. Psychol.* 13:873170. 10.3389/fpsyg.2022.873170 35519626 PMC9066092

[B64] Truong T. N. N. (2017). Understanding first year university students’ passivity via their attitudes and language behaviors towards answering questions in class. *Ho Chi Minh City Open Univ. J. Sci.* 7 84–93.

[B65] Truong T. N. N. Wang C. (2019). Understanding Vietnamese college students’ self-efficacy beliefs in learning English as a foreign language. *System* 84 123–132. 10.1016/j.system.2019.06.007

[B66] Tseng W. T. Dörnyei Z. Schmitt N. (2006). A new approach to assessing strategic learning: the case of self-regulation in vocabulary acquisition. *Appl. Linguist.* 27 78–102. 10.1093/applin/ami046

[B67] Vallotton C. Ayoub C. (2011). Use your words: the role of language in the development of toddlers’ self-regulation. *Early Child. Res. Quart.* 26 169–181. 10.1016/j.ecresq.2010.09.002 21969766 PMC3184006

[B68] Vanslambrouck S. Zhu C. Pynoo B. Lombaerts K. Tondeur J. Scherer R. (2019). A latent profile analysis of adult students’ online self-regulation in blended learning environments. *Comp. Hum. Behav.* 99 126–136. 10.1016/j.chb.2019.05.021

[B69] von Suchodoletz A. Trommsdorff G. Heikamp T. (2011). Linking maternal warmth and responsiveness to children’s self-regulation. *Soc. Dev.* 20 486–503. 10.1111/j.1467-9507.2010.00588.x

[B70] Vu D. V. Peters E. (2020). Learning vocabulary from reading-only, reading-while-listening, and reading with textual input enhancement: insights from Vietnamese EFL learners. *Region. Lang. Centre J.* 53, 85–100. 1–20. 10.1177/0033688220911485

[B71] Wang C. Bai B. (2017). Validating the instruments to measure ESL/EFL learners’ self-efficacy beliefs and self-regulated learning strategies. *TESOL Quart.* 51 931–947. 10.1002/tesq.355

[B72] Wang Y. Treffers-Daller J. (2017). Explaining listening comprehension among L2 learners of English: the contribution of general language proficiency, vocabulary knowledge and metacognitive awareness. *System* 65 139–150. em.2016.12.013 10.1016/j.syst

[B73] Webb S. (2007). The effects of repetition on vocabulary knowledge. *Appl. Linguist.* 28 46–65. 10.1093/applin/aml048

[B74] Webb S. (2013). “Depth of vocabulary knowledge,” in *The Encyclopedia of Applied Linguistics*, ed. Chapelle C. A. (Hoboken: Wiley Blackwell), 1656–1663. 10.1002/9781405198431.wbeal1325

[B75] Webb S. Nation P. (2017). *How Vocabulary is Learned.* Oxford: Oxford University Press.

[B76] Webb S. A. Sasao Y. (2013). New directions in vocabulary testing. *Region. Lang. Centre J.* 44 263–277. 10.1177/0033688213500582

[B77] Weiland C. Barata M. C. Yoshikawa H. (2014). The co-occurring development of executive function skills and receptive vocabulary in preschool aged children: a look at the direction of the developmental pathways. *Infant Child Dev.* 23 4–21. 10.1002/icd.1829

[B78] Wilang J. D. Duy T. V. (2021). Relationships of language learning variables in the acquisition of third languages in a multilingual context. *Int. J. Eval. Res. Educ.* 10 1117–1124. 10.11591/ijere.v10i4.21594

[B79] Yabukoshi T. (2021). Self-regulation and self-efficacy for the improvement of listening proficiency outside the classroom. *Lang. Learn. J.* 49 27–40. 10.1080/09571736.2018.1472626

[B80] Yanagisawa A. Webb S. (2020). “Measuring depth of vocabulary knowledge,” in *The Routledge Handbook of Vocabulary Studies*, ed. Webb S. (England: Routledge), 371–386.

[B81] Yang Y. (2016). *Frequency-based HSK Vocabulary.* China: Sinolingua.

[B82] Yerdelen S. Sungur S. (2019). Multilevel investigation of students’ self-regulation processes in learning science: classroom learning environment and teacher effectiveness. *Int. J. Sci. Mathe. Educ.* 17 89–110. 10.1007/s10763-018-9921-z

[B83] Yeşilbursa A. Bilican R. (2013). Validation of self-regulatory capacity in vocabulary learning scale in Turkish. *Procedia Soc. Behav. Sci.* 70 882–886. 10.1016/j.sbspro.2013.01.134

[B84] Zhang X. Lu X. F. (2015). The relationship between vocabulary learning strategies and breadth and depth of vocabulary knowledge. *Modern Lang. J.* 99 740–753. 10.1111/modl.12277

[B85] Zhou J. (2021). The contribution of morphological awareness and vocabulary knowledge to Chinese as a second language reading comprehension: a path analysis. *J. Psycholinguist. Res.* 51 55–74. 10.1007/s10936-021-09810-2 34536185

[B86] Zimmerman B. J. (2011). “Motivational sources and outcomes of self-regulated learning and performance,” in *Handbook of Self-Regulation of Learning and Performance*, eds Zimmerman B. J. Schunk D. H. (England: Routledge), 49–64.

[B87] Zimmerman B. J. Risemberg R. (1997). Becoming a self-regulated writer: a social cognitive perspective. *Contemp. Educ. Psychol.* 22 73–101. 10.1006/ceps.1997.0919

